# Utilizing knowledge base of amino acids structural neighborhoods to predict protein-protein interaction sites

**DOI:** 10.1186/s12859-017-1921-4

**Published:** 2017-12-06

**Authors:** Jan Jelínek, Petr Škoda, David Hoksza

**Affiliations:** Department of Software Engineering, Faculty of Mathematics and Physics, Charles University, Ke Karlovu 3, Prague 2, Czech Republic

**Keywords:** Protein-protein interaction, Prediction, Molecular fingerprints, Data mining

## Abstract

**Background:**

Protein-protein interactions (PPI) play a key role in an investigation of various biochemical processes, and their identification is thus of great importance. Although computational prediction of which amino acids take part in a PPI has been an active field of research for some time, the quality of in-silico methods is still far from perfect.

**Results:**

We have developed a novel prediction method called INSPiRE which benefits from a knowledge base built from data available in Protein Data Bank. All proteins involved in PPIs were converted into labeled graphs with nodes corresponding to amino acids and edges to pairs of neighboring amino acids. A structural neighborhood of each node was then encoded into a bit string and stored in the knowledge base. When predicting PPIs, INSPiRE labels amino acids of unknown proteins as interface or non-interface based on how often their structural neighborhood appears as interface or non-interface in the knowledge base. We evaluated INSPiRE’s behavior with respect to different types and sizes of the structural neighborhood. Furthermore, we examined the suitability of several different features for labeling the nodes. Our evaluations showed that INSPiRE clearly outperforms existing methods with respect to Matthews correlation coefficient.

**Conclusion:**

In this paper we introduce a new knowledge-based method for identification of protein-protein interaction sites called INSPiRE. Its knowledge base utilizes structural patterns of known interaction sites in the Protein Data Bank which are then used for PPI prediction. Extensive experiments on several well-established datasets show that INSPiRE significantly surpasses existing PPI approaches.

## Background

Protein interactions are crucial in a wide range of biological processes such as signal transduction or oxygen binding. Understanding interactions is thus important for revealing protein function. The knowledge of interactions can also be used in drug design as they play a key role in virtually all diseases.

Since experimental methods for protein-protein interaction (PPI) sites determination are time consuming and financially demanding, a great effort has been devoted to the development of computational methods of PPI identification. The purpose of these methods is, given a protein structure, to label surface amino acids that have the potential to be part of an interaction site with another protein. The obtained information can be subsequently used in the construction of PPI networks or simulated docking. Esmaielbeiki et al. [1] provided an overview of more than sixty methods for PPI prediction.

The existing methods can be grouped into three classes: evolutionary-based, template-based, and machine learning-based methods.

Evolutionary-based methods gain from the fact that evolutionary related proteins usually interact in the same manner and thus interaction sites have a higher degree of conservation to preserve their function. Furthermore, interacting pairs often co-evolve because changes in one interaction site are compensated by changes in the opposite interaction site in order to preserve their functionality [2].

Template-based methods require another protein (template) with known interaction sites. Since similar proteins interact in a similar way, the known interaction sites can be transferred to the new protein [3, 4]. The drawback of these methods is that they require a template protein which might not be always available.

Since the information required by evolutionary and template-based predictors is often not available, machine learning methods are commonly utilized. Machine learning methods pick appropriate characteristics to describe specific regions of a protein surface, which usually correspond to individual amino acids or their neighborhoods. A model is then trained on a set of positive and negative examples to recognize the values of characteristics and patterns commonly exhibited by PPIs. The trained model is subsequently used when an unknown protein needs to be characterized. A number of descriptors have been utilized for the purpose of PPI identification, such as hydrophobicity [5], energy of solvatation [6], propensity [5] or RASA (Relative Solvent Accessible Surface Area) [3–6], with RASA being especially popular [7]. As for machine learning approaches, the best performing methods utilize Support Vector Machines (SVM) [3, 5], Neural networks [8], Decision trees [6] or Conditional Random Fields (CRF) [9, 10].

CRF was one of the most recent machine learning methods applied for PPI prediction. It is a discriminative probabilistic undirected graphical model that can be considered as a Markov Random Field extended by a set of hidden (predicted) variables. The goal is to find the most probable labeling of hidden variables according to observations. Our approach was inspired by the CRF-based method presented by Dong et al. [9] and Wierschin et al. [10] where a protein is represented in a graph. In that representation, every amino acid corresponds to a node, and two nodes are connected by an edge if their corresponding amino acids are sufficiently close to each other. Amino acid descriptors (RASA in [9]) serve as observations in the CRF model, information about whether amino acids are parts of an interface or not translates into hidden variables, and transition probabilities need to be set in the training phase.

The idea behind CRF is to use transition probabilities to not allow situations where an amino acid would be labeled as interface but surrounded by non-interface amino acids only, i.e. a mislabeled amino acid; and vice versa. However, should an amino acid be surrounded by many mislabeled amino acids, CRF would not be able to repair it. In other words, CRF can be viewed as a kind of post-processing, smoothing the initial prediction. Therefore, the amino acids interface initial probabilities play a great role in CRF’s performance. Dong at al. [9] precomputed the initial probabilities of nodes for every RASA value according to a training dataset. In the prediction, initial probability for each node was set according to the RASA value of the corresponding amino acid. The drawback of such a method is that if two amino acids share the same RASA value they also have the same initial probabilities regardless of their neighborhood. But the neighborhood of an amino acid can have a significant influence on the interface state of that amino acid. Therefore, in [11] we outlined a possible approach which assigns initial probabilities based on the local neighborhood of an amino acid. It had many drawbacks and basically did not lead to an increased prediction ability and was meant rather as an illustration of the ability of graph databases to retrieve small graphs by means of subgraph isomorphism.

Here we introduce INSPiRE (INteraction Sites PREdictor) - a knowledge-based PPI prediction method that takes into account information about structural neighborhood of every amino acid and uses the idea of molecular fingerprints to efficiently store and query the knowledge base [12]. Although INSPiRE was originally inspired by [9], the current version outperforms existing approaches even without using CRF.

## Methods

The following list outlines the basic workflow of INSPiRE and the next sections detail the individual steps.

1. Retrieve protein-protein complexes from the Protein Data Bank [13].
2. Extract patterns representing local structural neighborhoods and interface/non-interface information for all the amino acids obtained in the previous step.
3. Convert the patterns into suitable data format for efficient storage and retrieval.
4. Label amino acids of unknown proteins as interface or non-interface based on how often their structural neighborhood appears as interface or non-interface in the knowledge base.

### Data retrieval

To build the knowledge base, we retrieved known complexes contained in Protein Data Bank (PDB) [13]. We used only complexes that consisted solely of proteins (no DNA or RNA fragments). PDB contains (as of November 2015) 60,743 such protein complexes. Next, we filtered out chains with less than five amino acids and subsequently filtered out complexes with less than two remaining chains. This resulted in 60,716 complexes having 220,555 chains with 54,204,183 amino acids. This data formed the basis for our knowledge base.

### Knowledge base construction

Protein structures in INPiRE are represented as labeled graphs the same way it was proposed in [9]. Amino acids correspond to nodes, and two nodes are connected by an edge if alpha-carbons of the corresponding amino acids are at most 6Å apart. Converting the data from the previous section into such graphs resulted in 292,938,242 edges, i.e. an amino acid had on average 5.4 neighbors.

An amino acid is labeled by INSPiRE as an interface amino acid if the van der Waals surface of at least one of its atoms is at most 0.5Å away from the van der Waals surface of any atom of another chain. According to this definition, 7,995,185 amino acids were labeled as interface and 46,208,998 amino acids were labeled as non-interface. Moreover, each node was labeled by a set of features which are later utilized in the prediction. Currently, INSPiRE uses two types of features:

- The type of amino acid (alanine, arginine etc.)
- RASA value, i.e. the fraction of a protein’s amino acids surface that is exposed to a solvent. This value was further binned into 10 unequal-sized bins. The size of bins was chosen so that each bin contained approximately 10% of amino acids in our knowledge base.

As mentioned above, INSPiRE uses patterns representing structure of amino acids’ local neighborhoods to discern interface and non-interface residues. Therefore, in the next step we extracted one subgraph for each node of every whole-protein graph. We call these subgraphs/patterns structural elements and we use two types of such elements:

- *d*_*i*_: Structural element consists of a central amino acid and all neighbors up to *i* edges from the central amino acid. In this case, the structural element is always a connected graph.
- *c*_*k*_: Structural element consists of a central amino acid and its *k*-nearest neighbors in 3D space. In this case, it can happen that the structural element is not a connected graph.

### Structural elements representation and storage

Since the knowledge base had to incorporate close to 55 millions structural elements, we needed an efficient way to store and retrieve the elements. Specifically, in the prediction phase we need for each structural element of the query protein to find out how many similar or identical structural elements are in the knowledge base. The problem of finding matching or similar elements translates into subgraph isomorphism which is NP-complete and is time demanding even for small graphs, which is our case. Obviously, querying a knowledge base consisting of millions graphs is a challenging task. We considered three possibilities for patterns encoding, storage and retrieval: graph data storage, relational data storage and molecular fingerprints stored in binary format.

Graph database allows one to natively store protein graphs and search for induced subgraphs defined by the query structural elements. We tried to adopt this approach in [11] where we used Neo4j graph database. Unfortunately, we found that this method is viable for structural elements only up to about 12 edges, but in our knowledge base approximately 45% of *d*_1_ structural elements have more than 12 edges and thus even for *d*_1_ the graph database is not an option.

Another possibility is to store the knowledge base in a relational DB. The natural representation would be to have one table for nodes and another table for edges. However, such representation leads to a lot of slow joins during every search for a given subgraph. A better way is to keep one table with nodes and precompute required information about its neighborhood, i.e. which features are present and how they are structured. Such information can then be stored in a string column and indexed using traditional indexing techniques. However, this is efficiently possible only for certain structural neighborhoods types. Specifically, we were able to implement so called radial pattern, where only the center and edges going from the center were taken into account. But adding also edges among the neighbors makes the problem much more challenging because several nodes can share a label, and more possibilities thus need be evaluated. From the retrieved records false positives need to be further filtered out using a specialized graph library. The filtration ratio of the database query is strongly dependent on the distribution of the employed feature types and often turned out to be quite weak. This poses a problem since the lower the filtration ratio the more time-consuming graph comparisons need to be done.

**Fig. 1:**
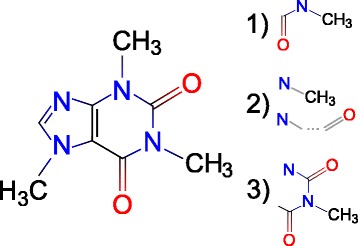
Examples of fragment types. 1) Linear fragment: paths of fixed length; 2) atom pair fragment: pairs of heavy atoms (adjacent or distant) along with the shortest path between them; 3) radial fragment: neighborhood within fixed number of bonds from the central atom

Although the combination of a relational DB and a specialized graph library can be applicable and provide reasonable results, its behavior is very dependent and sensitive to the distribution of the features. Therefore we took inspiration in molecular fingerprints traditionally used in virtual screening of small molecule libraries, an established component of drug discovery pipelines. Molecular fingerprints are a type of (lossy) representation of molecules as bit strings. The basic principle is to capture structural features of a molecular graph and encode them in a bit string which can be used later when assessing similarity to a pair of compounds. The advantage is that such representation is highly storage-efficient, and the time-consuming operation of comparison of two molecular graphs reduces to a highly time-efficient operation of bitstring comparison. There exists a wide variety of molecular fingerprinting methods which mainly differ in the type of topologies and physico-chemical features they encode [14–17]. Usually the entire molecule is not encoded all at once, instead it is fragmented into small parts called fragments (not necessarily disjunctive), and these fragments are encoded one by one. The most common types of fingerprints include encoding linear fragments (connected paths), dendritic fragments (trees), radial fragments (centered subgraphs), pairwise information (pairs of atoms that do not need to be neighbors), triplets, etc. [18]. Examples of fragment types are shown in Fig. 1.

To encode our structural elements, we decided to employ the Atom-Pairs fingerprint (AP) [14] which shows reasonable performance [17], and the main idea is relatively easy to implement. The outline of AP fingerprint construction follows:

1. Extract all atom pairs fragments
2. Encode fragments into integers (indexes)
3. Create a bitstring of length *n*
4. Hash the indexes into a space of the bitstring
5. For each hashed index turn on the corresponding bit, i.e. bits corresponding to atom pairs present in the molecule are turned on, the remaining bits are turned off

**Fig. 2:**
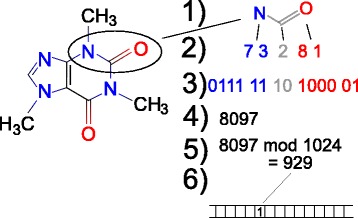
Construction of atom pair fingerprint. When creating an atom pair fingerprint, following steps are performed for each pair of heavy atoms: 1) extraction of given pair of atoms and the shortest path between them; 2) encoding of descriptors (atom type and the number of bonds for both atoms and their topological distance); 3) conversion into bit strings; 4) concatenation of bit strings into one number; 5) hashing the number into the index space; 6) setting the corresponding position in the fingerprint to 1

Besides this process, AP fingerprints also specify how fragments should be encoded into indexes. The idea is to consider the properties (in case of molecular fingerprints these are the number of bonds, atom type, etc.) and retrieve their values for each atom of a given fragment. These are then encoded into a limited number of bits (e.g. three bits are sufficient for bonds number) and assembled (via concatenation) to get the bit representation of the fragment index. The overall process outlines Fig. 2.

The AP construction process modified to our needs of encoding protein structural elements is as follows:

1. Construct fingerprint as a bit array *F* of length *l* and set all bits to 0
2. Iterate over all of amino acid pairs (*A*;*B*) in the structural element
  - Translate features of amino acids *A* and *B* in their codes $g_{i}^{A}$ and $g_{i}^{B}$ (for amino acid type, it is an order of its single letter code in a Latin alphabet; for RASA value, it is an index of the corresponding bin)
  - Determine the graph distance *d* of *A* and *B*
  - Concatenate $g_{1}^{A}$,..., $g_{n}^{A}$, *d*, $g_{1}^{B}$,..., $g_{n}^{B}$ (each represented as a binary number of a fixed length) into one number *i*
  - Set the (*i* mod *l*)-th element of *F* to 1

The resulting fingerprints, i.e. the encoded structural elements, can not be used directly to identify exact matches due to the employed hashing and because more amino acids can share a feature value and thus their stored images are ambiguous. Therefore, if an exact match was required, matched fingerprints would still need to be scanned for false positives. On the other hand, using fingerprints allows us to efficiently mine similar structural elements that are not exact matches. This is due to the fact that similarity of fingerprints and structural elements similarity correlate.

With an available reasonably efficient method for encoding structural neighborhoods, we took all the proteins, encoded structural neighborhood of each amino acid with the interface information and stored it in a binary file which formed the knowledge base to be used by INSPiRE in the prediction phase.

### PPI prediction

Once we have a knowledge base built we can use it to determine the probability whether a given amino acid of a given protein is part of an interface or not. The process consists of the following steps:

1. Create a graph for a given protein and label it with selected features (RASA value, amino acid type).
2. For each amino acid *A*:
  - Extract structural element *E* centered in *A*.
  - Pick out a subset *K*_*A*_ containing each element from the knowledge base, whose central residue has the same value of selected features as *A*.
  - Search *K*_*A*_ for *n* structural elements *S* most similar to *E*, where similarity is defined as the number of different bits in of the corresponding fingerprints.
  - Divide the retrieved structural elements into sets *I* and *N* based on whether their central amino acid is labeled as interface (set *I*), or non-interface (set *N*).
  - Use |*I*|/|*S*| as the probability of *A* being part of an interface.

## Results

In this section, we first evaluate the behavior of INSPiRE with respect to different parameters settings and then we compare it to the state-of-the-art methods. We used four datasets for evaluation; one dataset, called KL-subset [9], was used for training, while the other three datasets, PlaneDimers [5], TransComp1 [5] and DS188 [3], were used for testing. All experiments were carried out on a two Intel Xeon Processor X5660 (6 cores + hyper-threading) machine with 20 GB RAM. Since our knowledge base contained all the information from PDB, when searching for similar structural elements to a query all the query’s protein structural elements in the knowledge base were disregarded.

### Parameters tuning

To tune parameters of our method, we used the KL-subset defined by Dong et al. [9] which is a subset of a dataset published by Keskin et al. [19]. The dataset consists of 60 two-chain complexes, i.e. 120 proteins from which we excluded 2 complexes because they were protein-DNA complexes. The modified dataset thus consisted of 116 proteins.

To evaluate the quality of the model we used Matthews correlation coefficient (MCC) [20] which is the most commonly used measure to evaluate the quality of protein-protein interaction site predictors [7]. MCC is defined as

$$MCC = \frac{T_{P} * T_{N} - F_{P} * F_{N}}{\sqrt{(T_{P}+F_{P})(T_{P}+F_{N})(T_{N}+F_{P})(T_{N}+F_{N})}} $$

where *T*_*P*_ denotes the number of correctly labeled interface residues, *F*_*N*_ denotes the number of incorrectly labeled interface residues, *F*_*P*_ denotes the number of incorrectly labeled non-interface residues and *T*_*N*_ denotes the number of correctly labeled non-interface residues. The range of MCC is from -1 to 1, where 0 represents a random prediction, 1 is an absolutely correct prediction and -1 is the opposite of the correct prediction.

We measured the quality of prediction with respect to:

- Length of fingerprints
- Type of structural neighborhood and its size
- Considered features of amino acids in the structural elements (used for construction of fingerprints)
- Considered features of the central amino acid (used for prefiltering of the knowledge base)
- The number of most similar elements used for a prediction (if more elements were in the same distance, they were all considered)

#### Structural neighborhood

Although the *d*_*i*_ neighborhood (amino acids in given distance) seems to make more sense as chemical bonds have a delimited range and all structural elements cover approximately the same area in the *d*_*i*_ neighborhood, the *c*_*k*_ neighborhood (*k* nearest amino acids) shows better results in our tests (see Table 1). We ascribe it to the fact that the *c*_*k*_ neighborhood provides a more focused search because the probability of a structural element being in the knowledge base is dependent on the number of amino acids in the neighborhood. This can fluctuate significantly with the *d*_*i*_ type of neighborhood but not with the *c*_*k*_ neighborhood. A high fluctuation in the probability of an element being in the knowledge base leads to the situation where a knowledge base might not contain enough similar elements in a large part of queries, and simultaneously there might not be just one most similar element but a lot of equally similar elements in another set of queries.

**Table 1:** Comparison of different structural neighborhoods in terms of MCC

Surroundings	*c*_2_	*c*_4_	*c*_6_	*c*_8_	*c*_12_	*c*_16_	*c*_20_	*d*_1_	*d*_2_
MCC	0.090	0.475	0.643	0.670	0.685	0.682	0.674	0.548	0.555

(Fingerprints length: 1023 bits; features type: amino acid type only; one most similar element)

Another advantage of the *c*_*k*_ neighborhood is that it has higher granularity of steps than *d*_*i*_. When we focus on the number of nearest neighbors in the *c*_*k*_ neighborhood, we see an increase in prediction quality with a growing number of neighbors for *k* less than 12. It means that this increase adds a new piece of information that is useful for distinguishing between interacting and non-interacting amino acids. Although we expected a higher number of neighbors to decrease the prediction quality, as too remote and thus irrelevant residues are taken into account, we did not observe a significant decrease in the prediction quality even for *c*_20_ neighborhood, which covers 9% of an average protein.

#### Features types

**Fig. 3:**
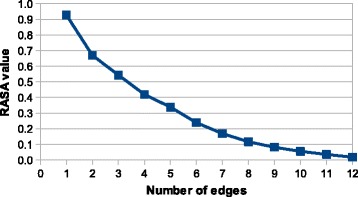
The relationship between RASA value and the number of edges. The figure shows the dependence of average RASA value of amino acids on the number of edges going from the corresponding nodes

When we focused on the features used to label the nodes, we saw a significant difference between the performance of the method when using an amino acid type and/or RASA value (see Table 2). Please note that we allow for a different feature type of the central node (which needs to match the query exactly) and the structural neighborhood. Surprisingly, using the RASA value only, gives the worst performance and also the combination of the RASA value with the amino acid type leads to worse results than using the amino acid type alone. This behavior has probably three reasons. First, more features result in a bigger index space leading to higher probability of collisions during hashing. A collision in a fingerprint means that two structural elements share the same position in the fingerprint and thus the most similar fingerprint might actually represent a different structural element. The second reason is related to the curse of dimensionality: more features result in higher probability that two similar structural elements have some different features and also that two non similar elements have some similar features. This leads to the decrease of the distance difference between similar and non-similar elements. Third, there is a strong correlation (− 0.83 according to Pearson’s correlation coefficient) between the RASA value and the number of edges leading from the residue (see Fig. 3), thus using the RASA value does not add sufficient amount of new information, and on the contrary, similar RASA values can be binned into different bins due to rounding.

**Table 2:** Comparison of different features in terms of MCC

		Central amino acid		
		aa	aa & rasa	rasa
Fingerprint	aa	0.643	0.641	0.620
	aa & rasa	0.518	0.567	0.535
	rasa	0.364	0.381	0.337

(Neighborhood: *c*_6_; fingerprints length: 1023 bits; one most similar element)

#### Number of most similar elements

**Fig. 4:**
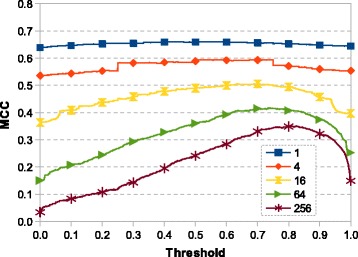
The relationship between prediction quality, threshold and number of most similar elements. The dependence of the prediction quality based on the number of most similar elements used for the prediction (individual lines) and on the threshold (X-axis). The threshold specifies the minimum portion of retrieved elements to be labeled as interface in order to denote the evaluated amino acid as an interface one. (Neighborhood: *c*_12_; fingerprints length: 1023 bits; features type: fingerprints with amino acid type and both amino acid type and RASA value on the central residue)

Next parameter we tested was the number of the most similar elements retrieved from the knowledge base based on which the interface probability of the query’s central node is computed. Generally, the less elements are taken, the more is the prediction affected by chance. On the other hand, taking too many neighbors can lead to bias since irrelevant elements are taken into account. Figure 4 shows that in case of predicting PPIs, decreasing the number of used similar elements leads to better results.

#### Fingerprints length

The longer the fingerprints are, the more time it takes to compare them. On the other hand, shorter fingerprints translate to a higher probability of hashing collisions and thus a higher probability of false positive matches. Specifically, when we increased the length from 63 to 255 bits, the time increased 3.9 times and MCC increased from 0.576 to 0.676. The change from 63 to 1023 bits lead to 8.6 time increase and MCC further increased to 0.685. In these experiments we used an amino acid type, the neighborhood was fixed to *c*_12_ and one most similar element was used for making prediction.

### Comparison with existing methods

After we tuned INSPiRE’s parameters, we compared it to the state-of-the-art methods used for prediction of protein-protein interaction sites.

As we mentioned in the introduction, there exists a multitude of methods for PPI prediction, but not all of them are available and tested on publicly available datesets. Therefore we chose six most often cited methods tested on public datasets.

In this section, the INSPiRE parameters were set as follows: *c*_12_ neighborhood, fingerprints length 1023 bits, the considered feature was amino acid only, one most similar element was used for prediction, and the threshold was 0.5175. The knowledge base was stored in a binary file taking up 6.66 GB. In a single-thread mode, the prediction took about 5 minutes per protein.

For comparison, we used PlaneDimers (127 proteins) and TransComp1 (100 proteins) datasets compiled by Zellner et al. [5] and DS188 dataset (188 proteins) compiled by Zhang et al. [3]. In PlaneDimers, protein complex with PDB ID 1O0Y became obsolete and we therefore replaced it with its actual version. From DS188 we excluded one chain (PDB ID 2HMI.A) because it comes from a protein-DNA complex. Moreover, DS188 contained three chains that were in the training dataset as well. For the PlaneDimers and TransComp1 datasets, surface residues were defined as those with *RASA*≥0.05 while for DS188 the rule was *RASA*>0.

Results showing the comparison of INSPiRE with SPPIDER [21], PresCont [5] and MetaPPISP [22] in terms of MCC on the PlaneDimers and TransComp1 datasets are in Table 3. The MCC values of the other methods are taken from [5]. The comparison with PredUs [4], PrISE [23], RAD-T [6] and MetaPPISP [22] are summarized in Table 4. Performance of those methods, which also includes precision, recall, accuracy and F1 measure, are borrowed from [3, 6, 23]. Both tables show that INSPiRE outperforms all of the state-of-the-art methods according to the MCC measure. Furthermore, INSPiRE is also better in the accuracy, F1 measure and precision on the DS188 dataset. PredUs and RAD-T have better recall, but they have worse precision which is understandable since precision and recall are intertwined values.

**Table 3:** Comparison on PlaneDimers & TransComp1 datasets in terms of MCC

	PlaneDimers	TransComp1
INSPiRE	**0.681**	**0.529**
SPPIDER [21]	0.330	0.150
PresCont [5]	0.330	0.170
MetaPPISP [22]	0.040	0.311

The best achieved value on each dataset is highlighted by boldface

**Table 4:** Comparison on the DS188 dataset

	MCC	Precision	Recall	ACC	F1
INSPiRE	**0.481**	**0.534**	0.567	**0.879**	**0.550**
PredUs [4]	0.345	0.503	0.575	0.726	0.530
PrISE [23]	0.338	0.480	0.432	0.806	0.455
RAD-T [6]	0.222	0.285	**0.647**	0.652	0.355
MetaPPISP [22]	0.262	0.490	0.267	0.811	0.346

The best achieved value in each metric is highlighted by boldface

## Discussion

What is surprising with regard to INSPiRE is that it works best with an amino acid type feature only and that this feature is not commonly employed, especially with regard to the simplicity of this feature. In contrast, the results of the widely used RASA feature are rather poor. To further explore why the amino acid type works so well in our case we focused on how INSPiRE differs from the existing methods that use information about local neighborhood or the propensity of an amino acid to be part of an interface. For example, PrISE computes the RASA value for a local structure neighborhood of an amino acid as a whole and also compares histograms of selected atom types in the neighborhood. PresCont utilizes the propensity of amino acid pairs to be a part of interface. But these approaches usually do not retain the information about the structure of a neighborhood; they utilize structural information only to identify the nearby residues.

**Fig. 5:**
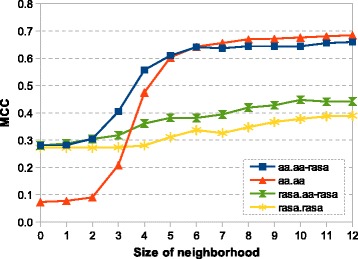
The relationship between prediction quality, size of the neighborhood and used features. The dependence of the prediction quality on the size of the used *c*_*k*_ neighborhood (X-axis) and on the used features (individual lines). Shown are the following features types: amino acid type only (AA.AA), fingerprints with amino acid type and both amino acid type and RASA value on the central residue (AA.AA-RASA), RASA value only (RASA.RASA) and fingerprints with RASA value and both amino acid type and RASA value on the central residue (RASA.AA-RASA). Fingerprints length was 1023 bits and one most similar element was used

**Fig. 6:**
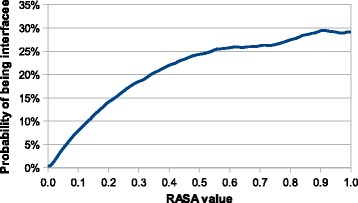
Probability of being an interface amino acid based on the RASA value. The dependence of probability to be an interface amino acid on the RASA value. For example an amino acid with RASA value less then 0.05 has at most 4% probability to be an interface, while an amino acid with RASA value higher than 0.5 has at least 24% probability to be an interface

INSPiRE is different in that it retains information about the structure of neighborhood. To confirm this, we disregarded information about the structural neighborhood and used the information about the central amino acid only, which is equivalent to *c*_0_ and *d*_0_ neighborhoods. The best result we were able to reach for amino acid type was *MCC*=0.078, while the RASA value reached *MCC*=0.272. It means that amino acid type itself corresponds to a virtually random predictor and the strength of this feature is based on using information about the neighborhood (see Fig. 5). In contrast to that, the RASA value itself is a better estimator of interface which can be explained by the fact that the amino acid must be on the surface to interact (see Fig. 6), but the improvement is not so significant when a bigger neighborhood is considered.

As we mentioned in the introduction, methods like CRF can be used in the final phase for smoothing the prediction. Thus we tried to utilize it for smoothing the prediction provided by INSPiRE. However, the better the prediction of INSPiRE was, the less improvement was achieved by utilizing CRF. E.g. *MCC*=0.523 was improved by CRF to *MCC*=0.560, while *MCC*=0.685 was improved only to *MCC*=0.687. This suggests that we almost completely exploit given information and new information must be added to improve the prediction quality.

In the chapter Structural elements representation and storage, we mentioned that the construction of fingerprints is ambiguous, i.e. two non-isomorphic graphs can have an identical fingerprint. In the case of settings used for comparison with the state-of-the-art methods, 4.8% of fingerprints in our knowledge base were ambiguous and 13% of residues in the knowledge base had an ambiguous fingerprint. Thus we tried to add an additional step to filter out non-isomorphic graphs with identical fingerprints. However, this filtration had no measurable effect on the prediction quality on the KL-subset (the difference was in the fourth decimal position) which indicates that in our case it is not necessary to specially treat hashing collisions in our case.

**Fig. 7:**
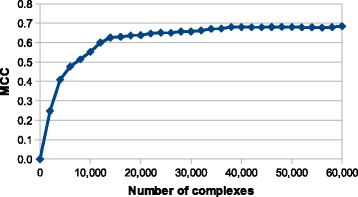
The relationship between prediction quality and size of the knowledge base. The figure shows the dependence of prediction quality on the number of complexes in the knowledge base. (Neighborhood: *c*_12_; fingerprints length: 1023 bits; features type: amino acid type only; one most similar element)

Finally, we asked ourselves whether a larger knowledge base with the same settings would increase the prediction quality or whether we had already reached the limits of the algorithm. To explore this, we created smaller subsets of the knowledge base used for comparison with the state-of-the-art methods based release dates of contained complexes. Results on the KL-subset showed that a subset of 13,000 complexes published before 2005 (21% of the full set) is enough to reach 90% of the prediction quality of the full knowledge base and that a subset of 38,000 complexes published before August 2011 (63% of the full set) differs in less then 1% of predictions (see Fig. 7). This suggests that further efforts should be focused on the quality control of complexes in the knowledge base instead of its enlargement.

## Conclusions

In this paper, we introduced INSPiRE a novel method for the prediction of protein-protein interaction sites. INSPiRE is a knowledge-based approach whose knowledge base is built over structural patterns in protein graphs of structures from the PDB. The knowledge base is utilized to search for amino acids with similar structural neighborhoods as the ones to be predicted as interface or non-interface. This was enabled by the utilization of molecular fingerprints, an approach widely used in virtual screening.

The prediction performance of INSPiRE significantly overcomes currently used methods on all tested datasets. We attribute the high performance to the utilization of not only the RASA value, but also of the amino acid type in combination with the preservation of information about the structural neighborhood arrangement of amino acids.
